# Treatment Success in Cancer: Industry Compared to Publicly Sponsored Randomized Controlled Trials

**DOI:** 10.1371/journal.pone.0058711

**Published:** 2013-03-21

**Authors:** Benjamin Djulbegovic, Ambuj Kumar, Branko Miladinovic, Tea Reljic, Sanja Galeb, Asmita Mhaskar, Rahul Mhaskar, Iztok Hozo, Dongsheng Tu, Heather A. Stanton, Christopher M. Booth, Ralph M. Meyer

**Affiliations:** 1 Center for Evidence-Based Medicine and Health Outcomes Research, Tampa, Florida, United States of America; 2 Department of Internal Medicine, Division of Evidence-Based Medicine and Health Outcomes Research, University of South Florida, Tampa, Florida, United States of America; 3 Moffitt Cancer Center & Research Institute, Departments of Hematology and Health Outcomes and Behavior, Tampa, Florida, United States of America; 4 Indiana University, Department of Mathematics, Gary, Indiana, United States of America; 5 NCIC Clinical Trials Group, Queen's University, Kingston, Ontario, Canada; 6 Departments of Community Health and Epidemiology, Queen's University, Kingston, Ontario, Canada; 7 Department of Oncology, Queen's University, Kingston, Ontario, Canada; Copenhagen University Hospital, Denmark

## Abstract

**Objective:**

To assess if commercially sponsored trials are associated with higher success rates than publicly-sponsored trials.

**Study Design and Settings:**

We undertook a systematic review of all consecutive, published and unpublished phase III cancer randomized controlled trials (RCTs) conducted by GlaxoSmithKline (GSK) and the NCIC Clinical Trials Group (CTG). We included all phase III cancer RCTs assessing treatment superiority from 1980 to 2010. Three metrics were assessed to determine treatment successes: (1) the proportion of statistically significant trials favouring the experimental treatment, (2) the proportion of the trials in which new treatments were considered superior according to the investigators, and (3) quantitative synthesis of data for primary outcomes as defined in each trial.

**Results:**

GSK conducted 40 cancer RCTs accruing 19,889 patients and CTG conducted 77 trials enrolling 33,260 patients. 42% (99%CI 24 to 60) of the results were statistically significant favouring experimental treatments in GSK compared to 25% (99%CI 13 to 37) in the CTG cohort (RR = 1.68; p = 0.04). Investigators concluded that new treatments were superior to standard treatments in 80% of GSK compared to 44% of CTG trials (RR = 1.81; p<0.001). Meta-analysis of the primary outcome indicated larger effects in GSK trials (odds ratio = 0.61 [99%CI 0.47–0.78] compared to 0.86 [0.74–1.00]; p = 0.003). However, testing for the effect of treatment over time indicated that treatment success has become comparable in the last decade.

**Conclusions:**

While overall industry sponsorship is associated with higher success rates than publicly-sponsored trials, the difference seems to have disappeared over time.

## Introduction

Randomized controlled trials (RCTs) are essential for discovery of new therapeutic interventions.[1] Research on evaluation of new cancer treatments in RCTs typically occur in the commercial and public sectors.[2] Whether commercial or public sector research programs generate higher rates of new successful treatments is not known.[2] Previous research based on analyses of publicly sponsored RCTs, indicate that about 25–50% of all new cancer treatments tested in RCTs are successful.[3] In comparison, industry-sponsored trials have been associated with success rates that are 45–50% higher, relative to publicly sponsored RCTs.[4]–[7] However, seemingly higher success rates in industry-sponsored trials could reflect bias (such as the choice of comparator) or the artefact of reporting (publication bias)[6]. This is because no study to-date took into account the correct assessment of both denominator representing the total number of all trials conducted by commercial and public funders and numerator, which refers to the number of “successful” results favouring the new treatment according to the primary outcome, which serves as the basis for trial design.[8], [9] Furthermore, difficulties to accurately assess the association of sponsorship and outcomes are also due to lack of the studies focusing on therapeutic advances in one field (e.g., cancer) and similar period of research development in industry and publicly supported studies.

In 2004, in settling a law-suit with the State of New York, the pharmaceutical company GlaxoSmithKline (GSK) agreed to set up a comprehensive online clinical trial registry making results from all trials, published or unpublished, publicly available.[10] This allowed systematic study of the pattern of treatment success in industry sponsored trials compared to publicly sponsored RCTs. In this paper, we compare the rate of therapeutic success of experimental versus standard therapies in RCTs involving cancer patients that were conducted by the NCIC Clinical Trials Group (CTG [previously National Cancer Institute of Canada Clinical Trials Group] for additional details on CTG see Supplementary material), an academic cancer clinical trials cooperative group that conducts national and international RCTs to those of GSK, the second largest, privately-funded pharmaceutical company.

## Methods

The assessment of treatment success of industry sponsored compared to publicly sponsored phase III cancer RCTs was performed according to the methods successfully applied in previous studies.[3], [11]–[13]

### Eligibility criteria

All consecutive, published and unpublished, phase III cancer RCTs assessing superiority of one treatment over another conducted by GSK or CTG and completed by June 2010 were eligible for inclusion. Since our main interest was to assess the proportion of RCTs in which experimental therapies were superior to existing treatments, we excluded from our analysis trials that had been designed to assess equivalence or non-inferiority rather than superiority. All RCTs from both cohorts cover the research development framework during the same time period (1980–2010).

### Data sources and study selection

A comprehensive list of all phase III RCTs along with associated protocols and publication citations was obtained from the CTG. For GSK, a detailed list of all phase III RCTs in cancer was created through a broad search of the GSK clinical trial registry available on the worldwide web.[14] All trial protocols from GSK and CTG were reviewed independently by two reviewers to determine their eligibility. The accuracy of the final list (denominator) of included studies was verified by CTG and GSK representatives.

### Data extraction

Two reviewers independently extracted data from eligible study protocols and publications using a standardised form.[3], [11]–[13] Published and unpublished studies were included in the final analysis. In the case of unpublished CTG studies, data were provided by the CTG. For unpublished GSK studies, data were extracted from trial summary reports. Data extracted included trial characteristics, publication status, type of publication, type of cancer, treatment details, treatment category, and choice of control intervention. Methodological quality domains relevant to minimising bias and random error for each included RCT were recorded according to established methods.[15]

### Assessment of treatment success

The superiority of experimental or standard treatment was assessed three ways [3], [11]–[13]:

1. each trial result was classified as statistically significant or not for the *a priori* specified primary outcome (favouring experimental or standard treatment) and final outcomes were summarised as proportions;
2. as statistical significance does not capture all subtleties involved in identifying treatment success or the trade-off between benefits and harms of competing interventions, we also assessed the success rate by determining the proportion of trials that according to investigators' judgments were deemed to be successful (assessed on a 6 point scale where a score of 1 favours the standard treatment and a score of 6 favours the experimental treatment). In cases where experimental treatments were judged by investigators to replace standard treatment as new standard of care, such experimental treatments were classified as “fit for adoption as standard of care”. Further details on assessment methods are provided in the supplementary material. These methods have been used successfully in previous studies by us with high reliability, face and content validity[3], [11]–[13] as well as by others[16]. Additional detail on assessment of success rate as per investigators' judgment is provided in supplementary material Figure S1;
3. as the first two methods are based on ‘vote counting’ and do not take into account the magnitude of effect, sample size, or time to event data, we also performed a quantitative synthesis of aggregate data from each study on primary and secondary outcomes to assess a distribution of outcomes favouring standard or experimental treatments as per methods recommended by The Cochrane Collaboration.[15]

A fundamental premise in the overall assessment of treatment success is that investigators make their “bets” regarding the superiority of one treatment over another before a trial is conducted.[3], [5],[11]–[13] The evaluation of success of new treatment depends on the researchers' beliefs of the effect of treatment on the primary outcome regardless of the type of treatments studied. That is, a similar distribution of treatment success should be observed for curative, adjuvant, palliative, etc. therapies regardless whether primary outcome was survival, disease-free survival, response rates, symptom relief and so on.[3], [5], [11]–[13] We, therefore, analysed all trials together. Nevertheless, we do report results according to the different categories as well other subgroups (see Results).

Intertwined with the assessment of treatment success is the question whether investigators can predict the results in advance with high likelihood. That is, although as a rule, the investigators always hope that new treatments would turn superior to established therapies [3], [11]–[13], [17], [18], we have previously hypothesized that, in an unbiased testing, the researchers cannot predict the results.[3], [5], [17]–[19] That is, sometimes the investigators' predictions will prove right, sometimes they may be wrong, and sometimes there would be no true difference between the experimental and controlled treatments. As a result, we would expect to observe about equal distribution in the proportion of trials in which experimental therapies are superior or inferior to standard treatments.[3], [5], [17]–[19]

### Sensitivity analyses

In addition to the analysis focusing on primary outcomes, which reflects researchers/funders' ‘best bet’ on expected success of tested treatments to assess the robustness of our findings, we conducted sensitivity analyses according to the methodological quality of trials (bias and random error), publication status, choice of control intervention as well as according to most important cancer outcomes, types of treatment, etc. (see Results).

### Statistical analyses

Differences in proportion of success (experimental versus standard) according to the statistical significance and investigators' judgment within each cohort of trials (GSK and CTG) were assessed using a two-sample binomial test. Using a standard approach, data on primary outcomes were meta-analyzed and reported as odds/hazard ratios (OR/HR) with 99% confidence intervals (CI) under a random-effects model.[15] These data typically included time-to-event (e.g. survival and event-free survival) and dichotomous data (e.g. response rate). In case the data on primary outcome were based on the continuous data, they were transformed into OR and pooled with the remaining data.[15] Test of interaction was performed to assess for the differences in treatment effects between the subgroups.[15]

### Assessment of the pattern of treatment success over time

To assess if the treatment success changed over time, we employed time series method and meta-regression. In time series analysis, we hypothesized that if treatment success affects one another, we would expect to see significant correlation between experimental treatments at time *t* and preceding times. If testing in each trial is independent of another, a ’white noise’ pattern with no significant autocorrelation in the analysis would be expected. Because time series analysis may miss a trend in outcomes over time due to various exogenous factors such as a shift toward selection of comparator, or increase in the sample size over time, we also performed a meta-regression using the year of study as a co-variate, and expressed data for change in the trend of treatment effect over each decade including testing for statistical interactions in treatment effects between two cohorts.[20]

All statistical analyses were performed using STATA software. This work is reported according to the PRISMA guidelines.[21]

## Results

### Trials and Treatment Characteristics

**Figure 1:**
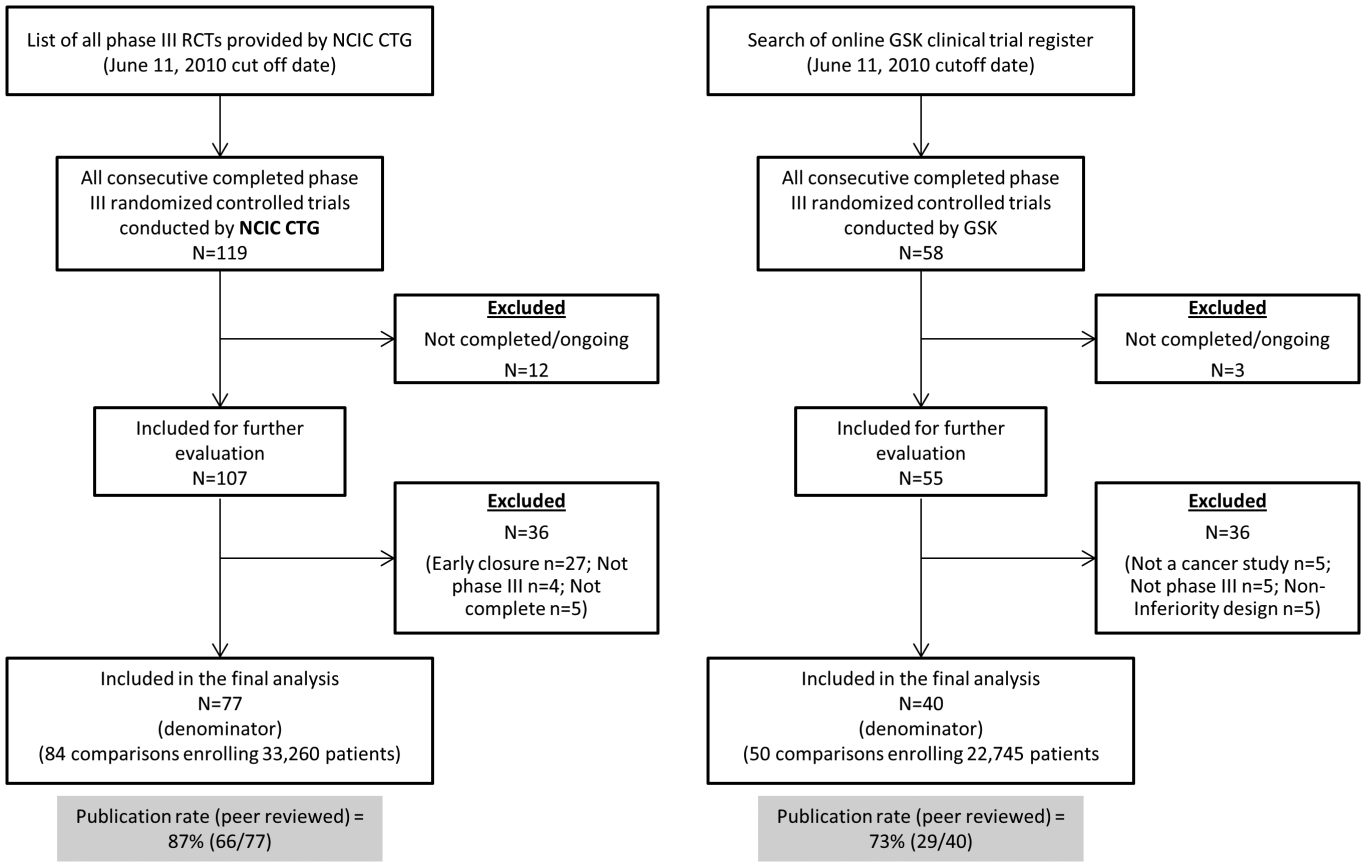
PRISMA flow diagram depicting process of identification and selection of studies.

Identification and selection of studies for GSK and CTG cohorts is illustrated in figure 1. The CTG cohort consisted of 77 trials (84 comparisons) enrolling 33,260 patients and GSK cohort consisted of 40 cancer RCTs (50 comparisons) which enrolled 19,889 patients. Trial characteristics of all included RCTs for CTG and GSK cohorts are summarized in Table 1. Data on primary outcomes were unavailable for 8 trials in the CTG cohort (see Figure 1). Survival was the primary outcome in 48% (40/84) of the CTG cohort and 6% (3/50) of GSK comparisons (p<0.0001). The most frequent type of treatment studied by CTG and GSK was in domain of palliative/supportive therapy; however, a difference in the percentages between two cohorts was significant (42% [35/84] in NCIC CTG vs. 84% [42/50] in GSK; p<0.001). Methodological quality of included studies for both cohorts is summarized in table 2. Overall, quality of trials conducted by CTG and GSK was good. However, there was a significant difference (at p<0.05) in the following risk of bias components: allocation concealment was adequately described in 100% of CTG trials (n = 84) compared to only 14% (7/50) of GSK studies; description of withdrawals/dropouts was provided in 75% (63/84) of CTG trials compared to 88% (44/50) of GSK studies. Similarly, blinding was described in 35% (29/84) of CTG trials, while 82% (41/50) of GSK trials reported it. Implications of the methodological quality on the overall results are further provided in the Discussion section.

**Table 1:** of included trials conducted by National Cancer Institute Canada Clinical Trials Group (CTG) and GlaxoSmithKline (GSK).

	NCIC CTG trials (N = 84 comparisons)	GSK trials (N = 50 comparisons)
	Number (%)	Number (%)
**Primary outcome**		
Survival	40 (48)	3 (6)
Event free survival	11 (13)	5 (10)
Tumor or antiemetic response*	29 (34)	42 (84)
Others	4 (5)	--
**Cancer type**		
Breast	12 (14)	4 (8)
Gynecologic	9 (11)	3 (6)
Hematologic malignancy	9 (11)	1 (2)
Lung	15 (18)	3 (6)
Prostate	3 (3)	1 (2)
Gastro-intestinal	4 (5)	--
Other types*	32 (38)	38 (76)
**Treatment category**		
Induction, First Line, Definitive/Curative	17 (20)	5 (10)
Consolidation	1 (1)	--
Adjuvant	19 (23)	--
Maintenance	9 (11)	--
Supportive/Palliative*	35 (42)	42 (84)
Other	3 (3)	3 (6)
**Treatment subcategory**		
Chemotherapy*	32 (38)	7 (14)
Endocrine	2 (2)	1 (2)
Radiation	6 (7)	--
Immunotherapy*	5 (6)	--
Combined	9 (11)	3 (6)
Targeted therapy	4 (5)	2 (4)
Anti-emetics*	14 (17)	37 (74)
Other	12 (14)	--
**Study design**		
Parallel	72 (86)	46 (92)
Cross-over	4 (5)	4 (8)
Factorial	8 (9)	--
**No. of comparisons**		
2 arms*	67 (80)	32 (64)
≥3 arms*	17 (20)	18 (36)
**Type of control**		
Another active	66 (79)	39 (78)
Placebo or no treatment	18 (21)	11 (22)

* A statistically significant difference exists between GSK and NCIC CTG cohorts (Fisher exact test P-values<0.05).

**Table 2:** Methodological quality of included trials conducted by National Cancer Institute Canada Clinical Trials Group (CTG) and GlaxoSmithKline (GSK).

	NCIC CTG trials (N = 84 comparisons)	GSK trials (N = 50 comparisons)
	Number (%)	Number (%)
**Risk of Bias**		
**Generation of randomization sequence**		
Computer generated	33 (39)	24 (48)
Not reported	51 (61)	26 (52)
**Allocation Concealment** *		
Adequate (central)	84 (100)	7 (14)
Not reported/unclear	0 (0)	43 (86)
**Description of withdrawals/dropouts** *		
Yes	63 (75)	44 (88)
No	21 (25)	6 (12)
**Blinding** *		
Yes	29 (35)	41 (82)
No/unclear	55 (65)	9 (18)
**Intention-to-treat analysis for benefits**		
Yes	68 (81)	38 (76)
No	16 (19)	12 (24)
**Per protocol analysis for adverse events** *		
Yes	25 (30)	4 (8)
No/unclear	59 (70)	46 (92)
**All pre-specified outcomes reported** *		
Yes	62 (74)	7 (14)
No	22 (26)	43 (86)
**Risk for random error**		
**Expected difference in primary outcome pre-specified**		
Yes	82 (98)	41 (82)
No	2 (2)	9 (18)
**Alpha/Beta level pre-specified**		
Yes	80 (95)	46 (92)
No	4 (5)	4 (8)
**Sample Size calculations performed ** ***a priori***		
Yes	82 (98)	48 (96)
No	2 (2)	2 (4)

* A statistically significant difference exists between GSK and NCIC CTG cohorts (Fisher exact test P-values <0.05)

### Evaluation of Treatment Success

**Figure 2:**
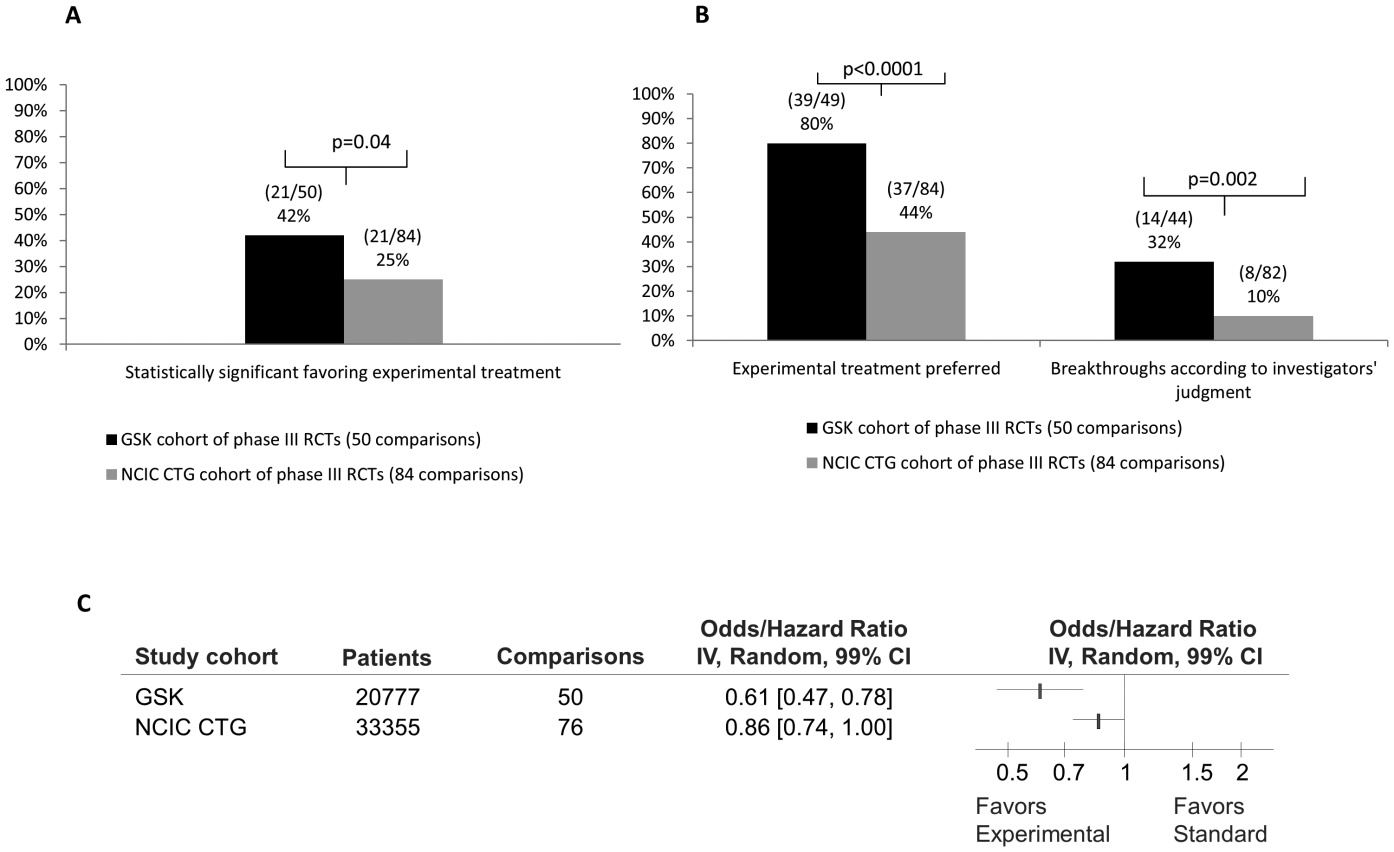
Success rate of GlaxoSmithKline (GSK) compared with National Cancer Institute Canada Clinical Trials Group (CTG) cohort of studies. (A) Distribution of success rate according to statistical significance of the results for the primary outcome; (B) Distribution of success rate according to investigators' judgments. *Data for one comparison in the GSK cohort were not available to make a decision on investigators' judgments. For ten comparison in the GSK cohort and two comparisons in the CTG cohort data were not available to make a judgment on whether investigators considered the experimental treatment to be a breakthrough ( = fit for adoption as standard of care”). **The results were available in the summary format (unpublished). Therefore, investigator judgments were not possible to assess for 10 comparisons. (C) Forest plot showing quantitative pooling of data on primary outcome for studies conducted by CTG and GSK. The summary pooled estimate (odds/hazard ratio) is indicated by rectangles, with the lines representing 99% confidence intervals (CIs).

**Figure 3:**
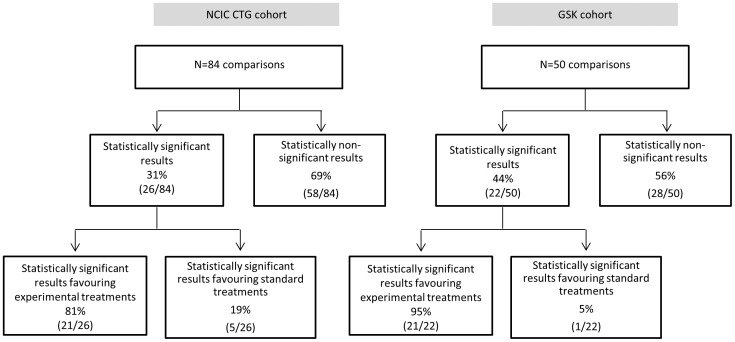
Distribution of success rate according to the results being statistically significant versus non-significant for the a priori specified primary outcome.

Figure 2 shows the success rate of GSK and CTG cohorts according to statistical significance (Figure2A), investigators' judgments (Figure.2B) and quantitative synthesis (Figure2C). As detailed in figure 3, the results were statistically significant in 44% (99%CI 26 to 62; 22/50) of GSK compared to 31% (99%CI 18 to 44; 26/84) of CTG trials(RR = 1.42; p = 0.128). However, in the GSK cohort, 42% (99%CI 24 to 60; 21/50) of the results that were statistically significant favoured experimental treatments compared to 25% (99%CI 13 to 37; 21/84) in the CTG cohort (RR = 1.68; p = 0.04) (Figures 2A & 3). As per investigators' judgments, new treatments were favoured over standard in 80% (99%CI 65 to 94; 39/49) of GSK trials compared to 44% (99%CI 30 to 58; 37/84) in the CTG studies (RR = 1.81; p<0.0001) (Figure 2B). The GSK investigators deemed 32% (99%CI 14 to 50; 14/44) of interventions as “fit for adoption as standard of care” compared to 10% (99%CI 1 to 18; 8/82) by CTG investigators (RR = 3.3; p = 0.002) (Figure 2B).

**Figure 4:**
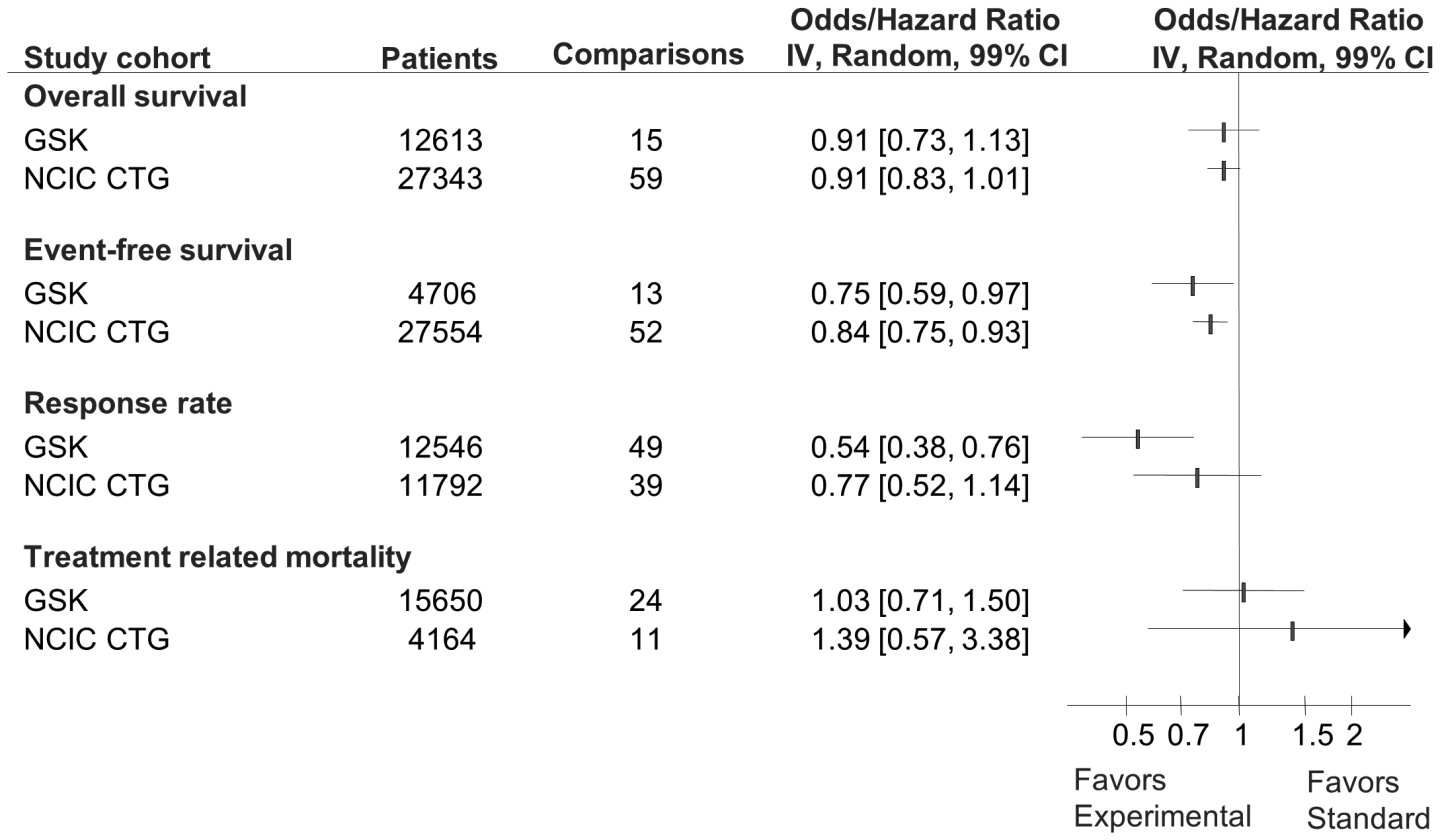
Forest plot of distribution of success rate for outcomes of overall survival, event-free survival, response rate, and treatment relate mortality for GlaxoSmithKline (GSK) and National Cancer Institute Canada Clinical Trials Group (CTG) cohorts. The summary pooled estimate (odds/hazard ratio) is indicated by rectangles, with the lines representing 99% confidence intervals (CIs). Note that unlike for the pooled analysis for all primary outcomes (Fig 2) test of interaction detected no statistically significant difference between subgroups, but the number of comparisons was too small to detect a difference between two cohorts.

**Figure 5:**
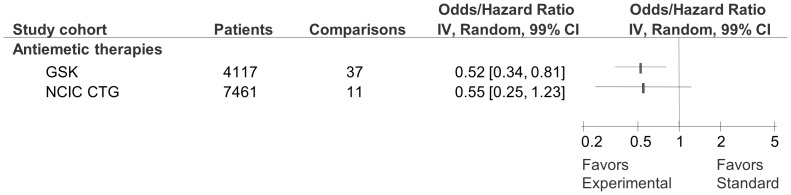
Forest plot of pooled data on primary outcome for studies of antiemetic therapies conducted by GlaxoSmithKline (GSK) and National Cancer Institute Canada Clinical Trials Group (CTG). Given the prevalence of studies involving antiemetic treatments, we include this subgroup analysis. The summary pooled estimate (odds/hazard ratio) is indicated by rectangles, with the lines representing 99% confidence intervals (CIs). The test of interaction is statistically non-significant between the two cohorts but the number of trials was small.

Meta-analysis of the primary outcomes indicated that the intervention effect was larger in GSK trials (as indicated by lower OR/HR in morbidity/mortality) than in the CTG cohort (OR/HR = 0.61 [99%CI 0.47 to 0.78] compared to 0.86 [99%CI 0.74 to 1.00]; p = 0.003 for test of interaction between two subgroups; Figure 2C). The results for other important outcomes are shown in figure 4. For the outcome of overall survival, the results showed that success rate of GSK cohort was similar to CTG and new treatments are as likely to be inferior or superior to standard treatments (OR = 0.91 [99%CI 0.73 to 1.13] compared to 0.91 [99%CI 0.83 to 1.01]; p = 1.00 for test of interaction between two subgroups). New treatments were slightly favoured for event free survival in both GSK (OR = 0.75, [99%CI0.57–0.97]) and CTG (OR = 0.84 [99%CI 0.75 to 0.93] cohorts but the test of interaction between the subgroups was not significant (p = 0.28; figure 4). For the outcome of response rates new treatments were favoured over standard treatments in the GSK cohort (OR = 0.54 [99%CI 0.38 to 0.76]) but not for the CTG cohort (OR = 0.77 [99%CI 0.52 to 1.14; p = 0.08 for test of interaction between two subgroups; figure 4). For the outcome of treatment related mortality new treatments were as likely to be superior or inferior to standard treatments for GSK (OR = 1.03 [99%CI 0.71 to 1.50]) and CTG cohort (OR = 1.39 [99%CI 0.57 to 3.38]; p = 0.423 for test of interaction; figure 4). Similarly, restricting analysis to anti-emetics therapy only (which represented the largest category of treatment in GSK and the second largest in the CTG research portfolio), we found similar treatment effects with no differences between two cohorts (OR = 0.52 [99%CI 0.34 to 0.81] compared to 0.55 [99%CI 0.25 to 1.23]; p = 0.873; figure 5).

**Figure 6:**
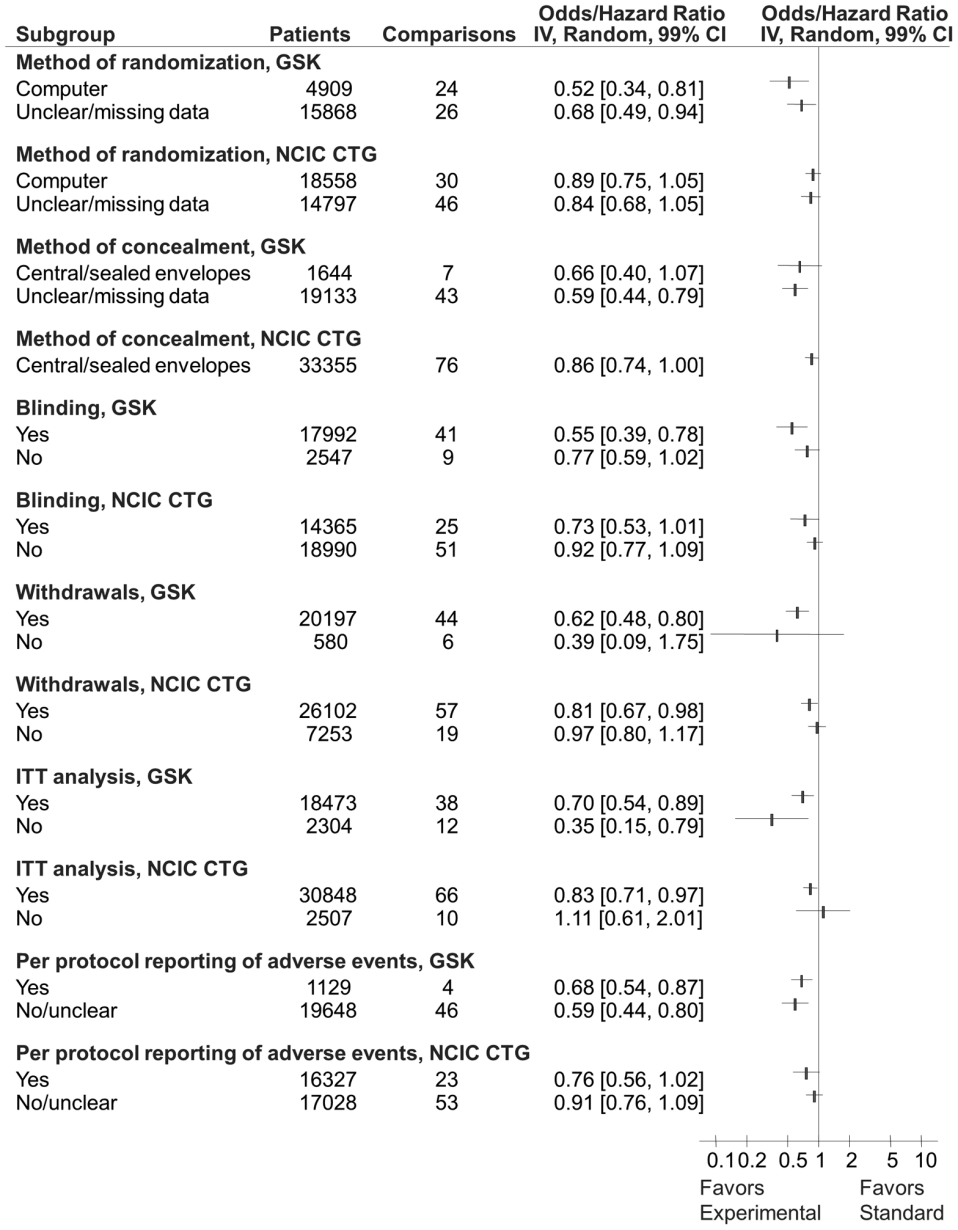
Forest plot of sensitivity analysis for distribution of success rate according to methodological quality of included studies for GlaxoSmithKline (GSK) and National Cancer Institute Canada Clinical Trials Group (CTG) cohorts. The summary pooled estimate (odds/hazard ratio) is indicated by rectangles, with the lines representing 99% confidence intervals (CIs).

**Figure 7:**
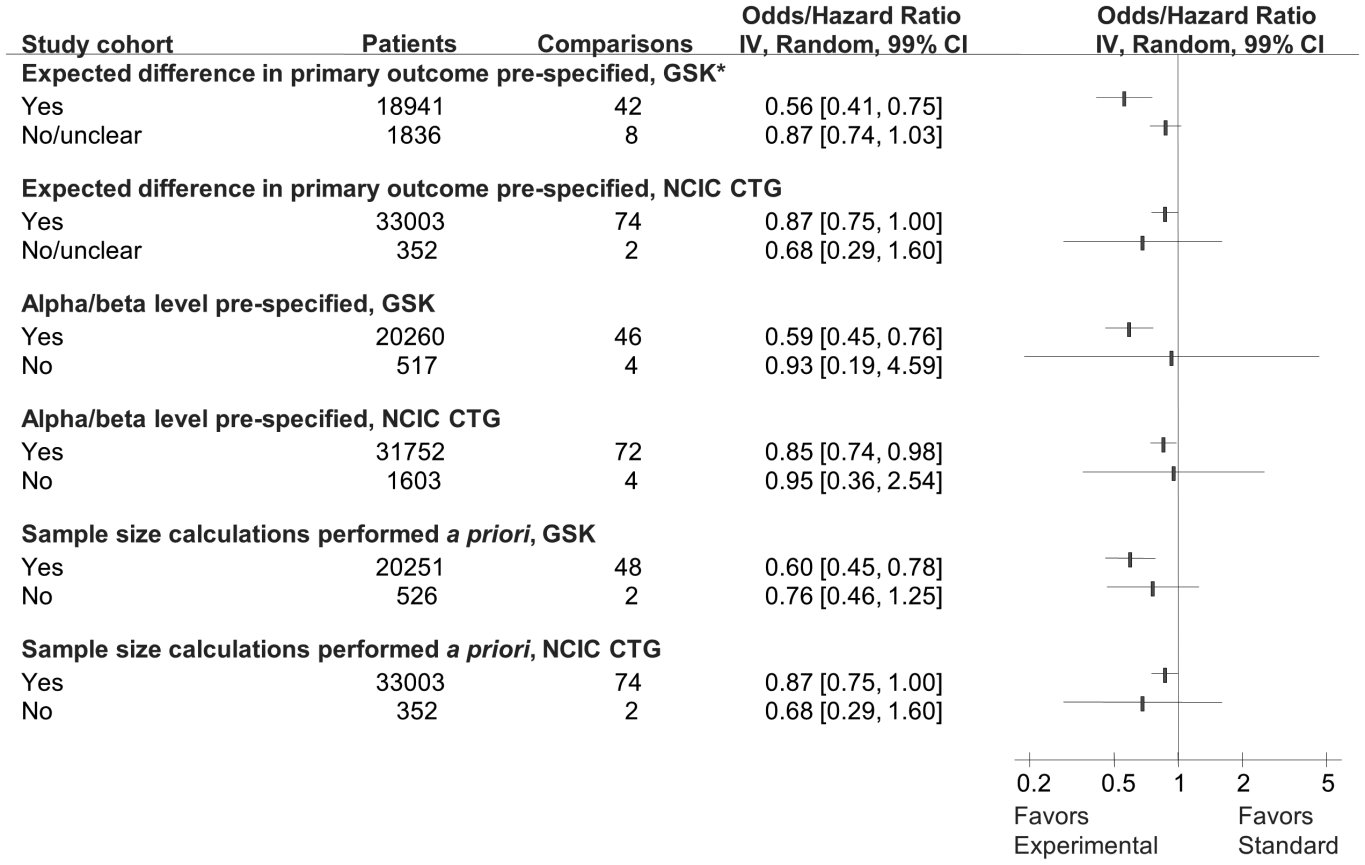
Forest plot of sensitivity analysis for distribution of success rate according to elements of random error that can potentially impact outcomes for GlaxoSmithKline (GSK) and National Cancer Institute Canada Clinical Trials Group (CTG) cohorts. The summary pooled estimate (odds/hazard ratio) is indicated by rectangles, with the lines representing 99% confidence intervals (CIs). * Represents a statistically significant test for interaction between subgroups. The test for interaction was statistically significant in cohort of GSK trials reporting versus not reporting expected difference for primary outcome.

**Figure 8:**
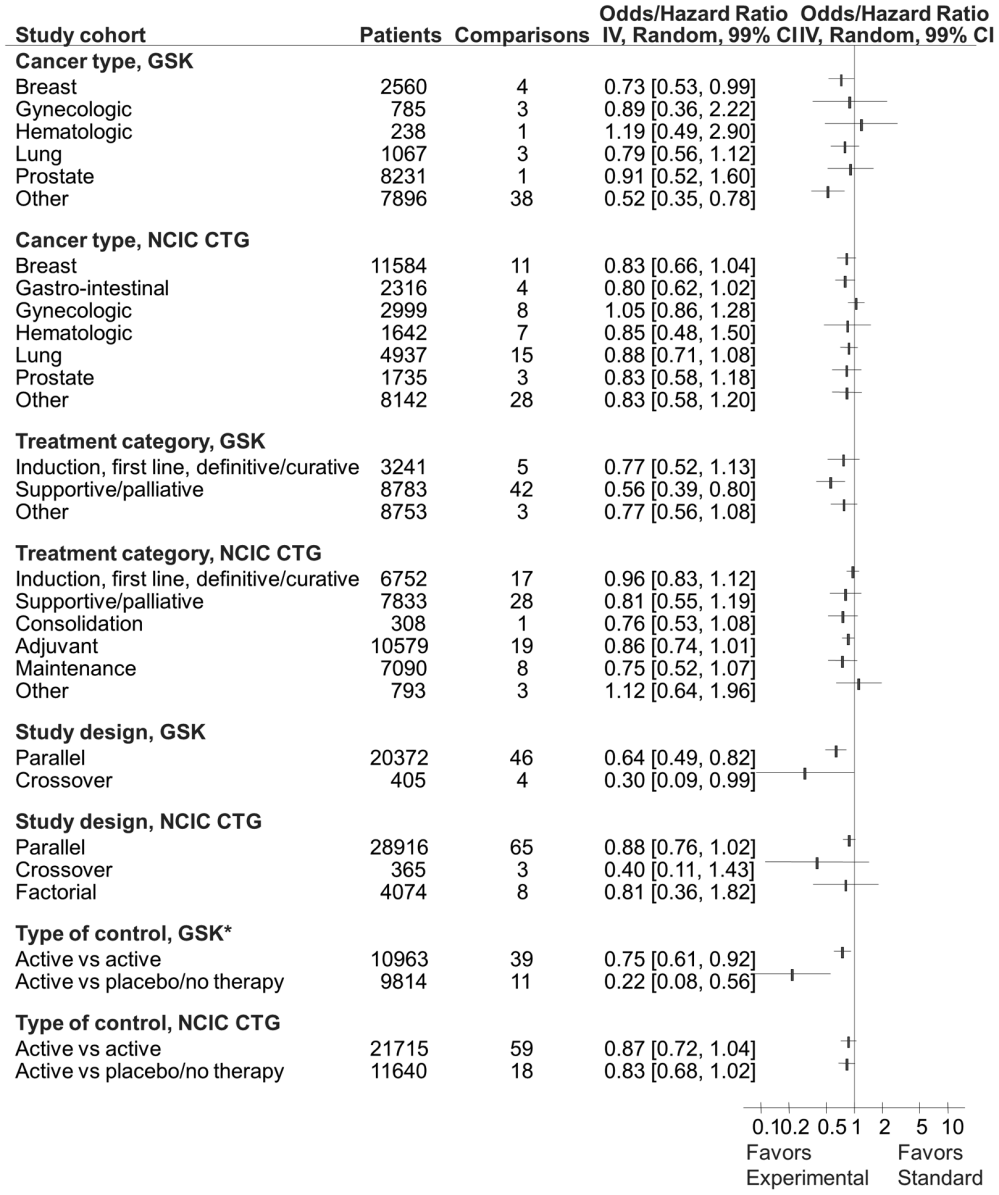
Forest plot of sensitivity analysis for distribution of success rate according to cancer and treatment type, study design, and choice of control for GlaxoSmithKline (GSK) and National Cancer Institute Canada Clinical Trials Group (CTG) cohorts. The summary pooled estimate (odds/hazard ratio) is indicated by rectangles, with the lines representing 99% confidence intervals (CIs). * Represents a statistically significant test for interaction between subgroups. Note that the experimental treatments were statistically superior in GSK trials where comparators were placebo or no therapy.

**Figure 9:**
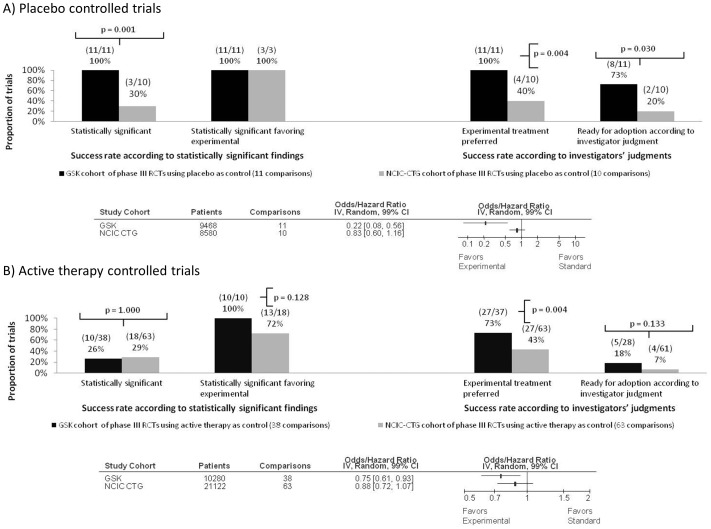
Comparison according to placebo versus active comparator. (A) Success rate according to statistically significant findings, investigator judgment and meta-analysis in placebo controlled trials. (B) Success rate according to statistically significant findings, investigator judgment and meta-analysis in trials having active treatment as control. Statistically significant results were observed in GlaxoSmithKline (GSK) trials according to all 3 metrics of summarizing treatment success (a proportion of statistically significant results favouring experimental treatment, according to the investigators' judgments and quantitative pooling of data). (B). When the active comparator was used, this was only true for the metrics according to investigators' judgments. Test of interactions in placebo-controlled trials was highly significant in favour of GSK compared to National Cancer Institute Canada Clinical Trials Group (CTG) comparisons (p = 0.001) while it was not significant (p = 0.154) between trials when active control was used as a comparator.

Sensitivity analyses according to risk of bias, type of cancer, treatment categories, and trial design are show in figures 6 through 8. The results from the sensitivity analyses showed that overall success rate of experimental versus standard treatments for GSK and CTG cohorts were not significantly impacted by risk of bias (Figure 6 and 7) despite the differences in risk of bias elements within and between the two cohorts (see table 2). Result of additional sensitivity analysis is reported in the supplementary material (Figure S2). As shown in figure 8, while GSK and CTG employed placebo/no therapy as a comparator in equal proportions, the effect size in the GSK trials employing placebo/no therapy as a comparator was significantly more pronounced than in the CTG trials (see Table 1). Similarly, 100% (11/11) of trials that employed placebo in GSK were statistically significant compared to 30% (3/10) of trials in CTG cohort (p = 0.001) (Figure 9).

**Figure 10:**
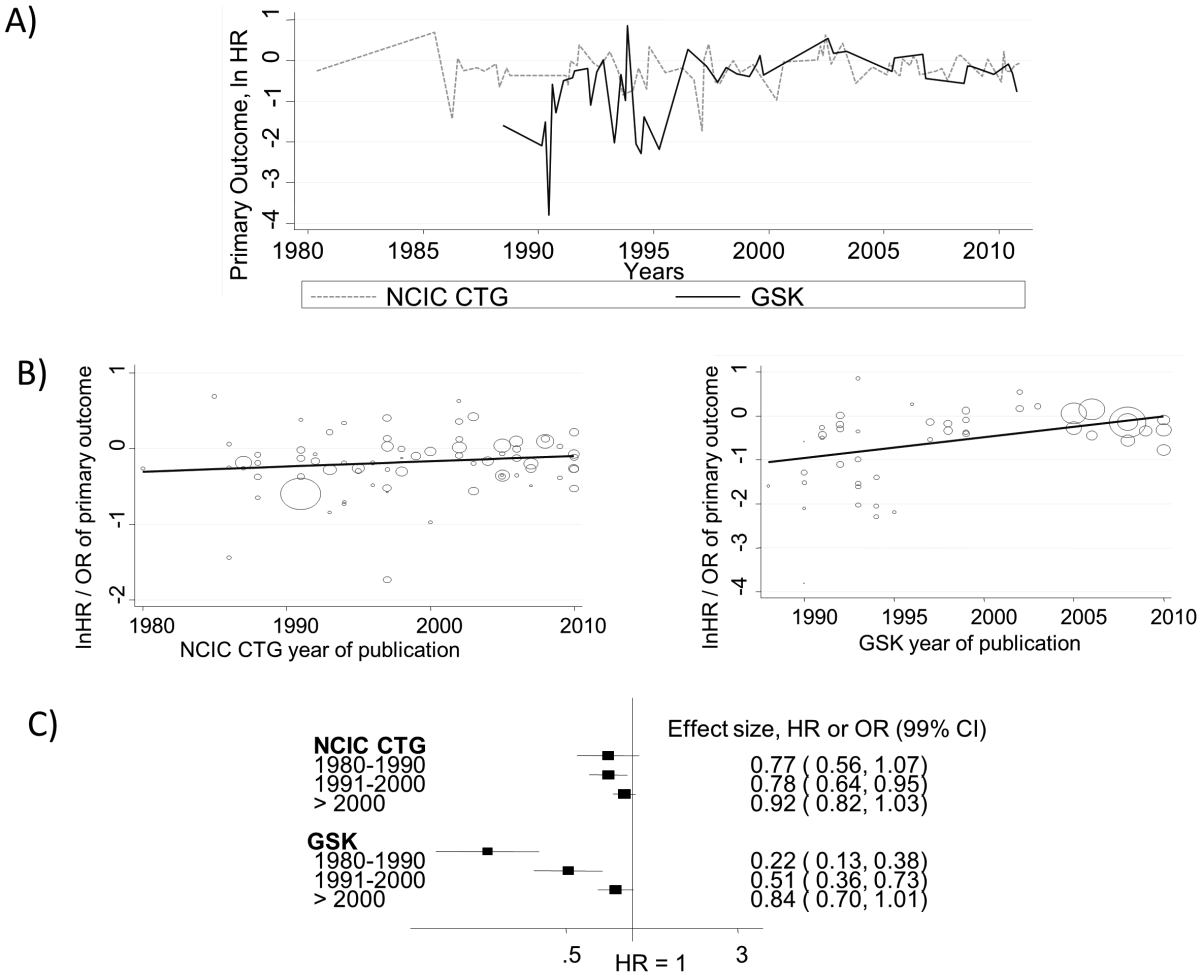
Assessment of the pattern of treatment successes over time. A) Time series analysis of treatment effect (natural logarithm of hazard ratio [ln HR]). Data are consistent with “white noise” pattern indicates no significant autocorrelation between studies carried out at various time intervals. An ln HR less than 0 indicates superiority of new treatments; greater than 0, superiority of standard treatments. B) Meta-regression analysis. The results shows a significant trend toward HR = 1 (logHR = 0) over time in the GlaxoSmithKline (GSK) cohort. That is, the average effect size of treatments decreased over time [by 38% per decade ( = exp (0.48) = 1.62)] For the National Cancer Institute Canada Clinical Trials Group (CTG) cohort there was no statistically significant change in the treatment effect over time. R^2^ = 24.48% for GSK i.e. the model accounted for about 25% of the observed variation in the results while R^2^ was virtually zero in the CTG cohort. C) Meta-analysis stratified according to time periods. The results confirm the findings of meta-regression. Vertical lines indicate lines of no difference between new and standard treatments. Note that a “no difference” result can be obtained when treatments are truly identical, or when experimental treatments are as successful as standard treatments (i.e., sometimes new treatments are better and sometimes standard treatments are better). Squares indicate point estimates. Horizontal lines represent 99% confidence interval (CI).

Figure 10 shows the effect of treatment over time. Time series analysis was consistent with the ‘white noise’ pattern indicating that each trial addressed the question independent of the preceding one. However, a meta-regression shows a significant trend toward HR = 1 (logHR = 0) over time in GSK cohort. That is, the average treatment effects decreased over time by 48% per decade (Figure 10B). For CTG cohort, there was no change in the magnitude of effect size over time. Coefficient of determination (R^2^) was 24.5% in the GSK analysis, i.e. the model accounted for about 25% of the observed variation in the results while R^2^ was virtually zero in the CTG cohort.

**Figure 11:**
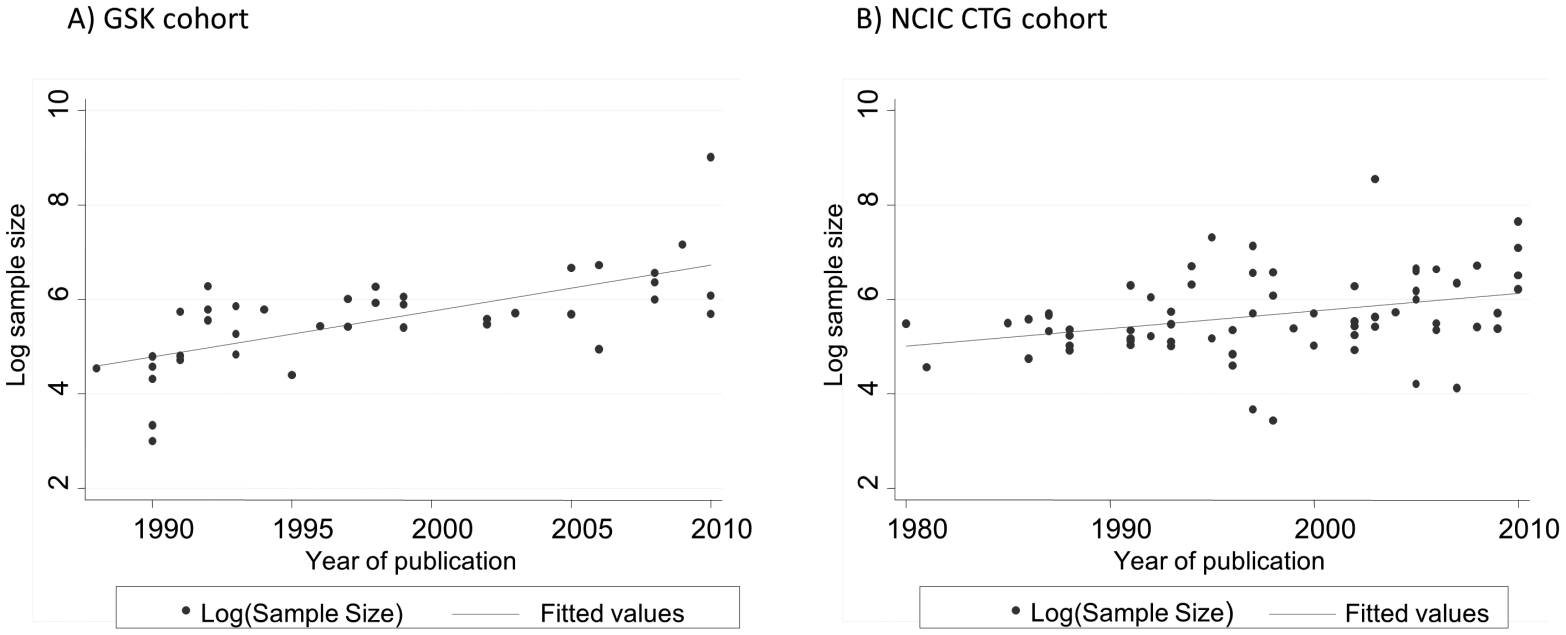
Change in sample size over time. There was an increase in the sample size over time both for National Cancer Institute Canada Clinical Trials Group (CTG) and GlaxoSmithKline (GSK) cohort. For CTG, on average, the sample size increased by 50% per decade, while for GSK, on average, it doubled. GSK trials were also somewhat larger than CTG trials: [median (range): 296 (20 to 8231) vs. 244 (31 to 5157); p = 0.062]. Sample size was somewhat larger in GSK trials [median (range): 296 (20 to 8231) compared to 244 (31 to 5157); p = 0.062]. It also increased over time in both cohorts. For CTG, on average the sample size increased by 50% per decade, while for GSK, on average, it doubled per decade. The average sample size increase (i.e. slope) was statistically significant between two cohorts (P<0.001).

Sample size was somewhat larger in GSK trials [median (range): 296 (20 to8231) compared to 244 (31 to 5157; p = 0.062). It also increased over time in both cohorts. For GSK, on average the sample size doubled per decade. For CTG, on average, it increased by 50% per decade. The average sample size increase (i.e. slope) was statistically significant between two cohorts (P<0.001) (Figure 11).

**Figure 12:**
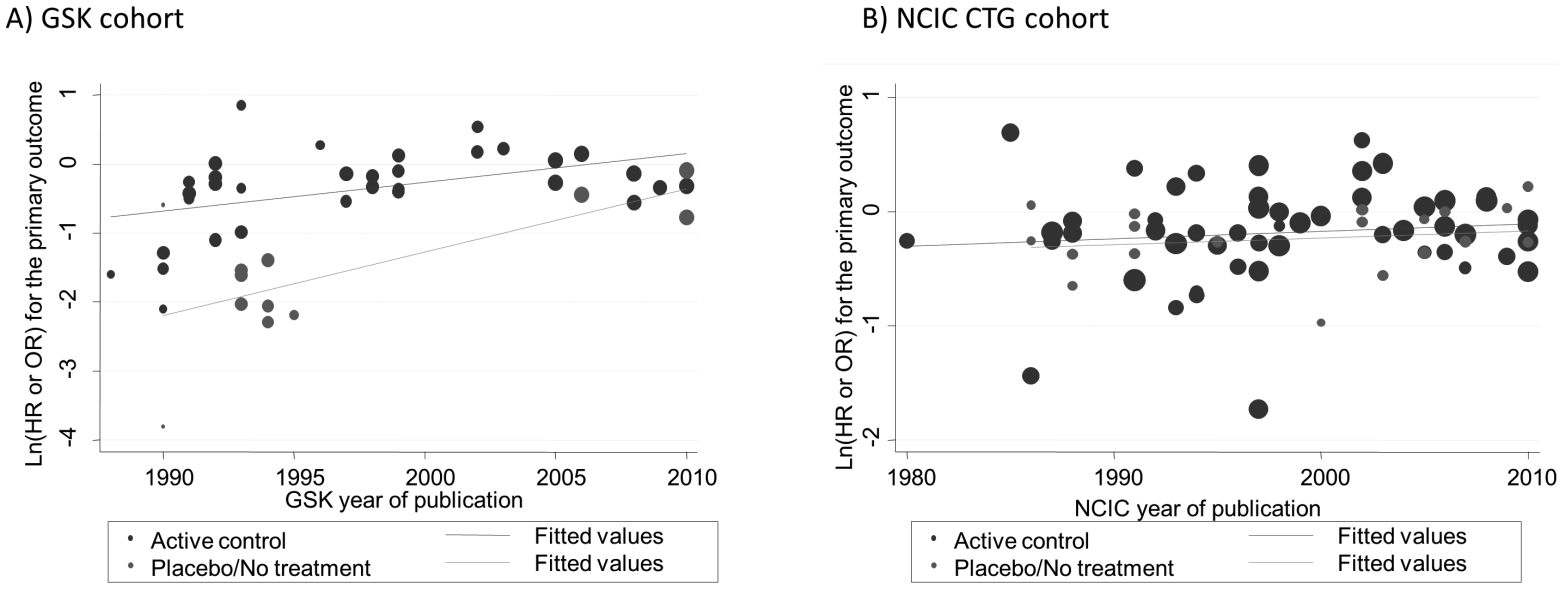
Meta-regression of effect of time (year of publication), choice of active control and sample size on the magnitude effect in National Cancer Institute Canada Clinical Trials Group (CTG) cohort of trials (A) and GlaxoSmithKline (GSK) cohort (B). None of the variables were statistically significant in NCIC CTG cohort of trials (R^2^ = −0.68%). In GSK cohort sample size showed no statistical significant association with the results (p = 0.08) while year (p = 0.048) and the choice of comparator (p<0.000) were statistically associated with the observed results in GSK cohort. These two variable accounted for about 72% of the observed variation in the results (R^2^ = 71.69%). In general, the effect size was closer to 1 (ln HR = 0) when the active comparator was employed.

To investigate whether the change in the use of comparator (active vs. placebo/no treatment), and increase in sample size can explain the change of the magnitude of treatment effect over time, a meta-regression using time, comparator and sample size was performed. The results show that none of these variables affected the results in the CTG cohort (R^2^ = −0.68%), which remained stable over three decades of observations. However, in GSK cohort, time (year of publication; p = 0.048) and the choice of comparator (p<0.0001) were associated with statistically significant effect on the effect size while sample size showed no such association (p = 0.08; Figure 12). These two variable accounted for about 72% of the observed variation in the results (R^2^ = 71.7%). Additional sensitivity analysis results from meta-analysis for distribution of success rate for trials involving multiple comparisons is illustrated in supplementary material Figure S2.

## Discussion

While other studies evaluated a relationship between sponsorship and health outcomes[5], [6], [22], no study specifically examined the question how often new treatments are superior to established treatment as a function of funding source. We present the first study evaluating the treatment success and pattern of therapeutic discoveries in industry sponsored versus publicly sponsored research. Using three metrics for assessment of therapeutic success, we showed that success rates differ between industry and publicly sponsored trials. Experimental treatments were, on average, favoured in industry sponsored research. New treatments resulting in statistically significant results were favoured more often in the GSK than in the CTG cohort (42% versus 25%; p = 0.04). Similarly, assessment of research success according to investigators' judgments demonstrated that experimental treatments were superior to standard treatments in 80% of GSK compared to 44% of NCIC CTG trials (p<0.001). Finally, quantitative pooled analysis of data for the primary outcome also indicated that success rates of GSK trials was superior to those of CTG trials (odds ratio = 0.61 compared to 0.86; p = 0.003). Thus, depending on the metric used, industry-sponsored trials are associated with 17 to 25% statistically significant greater rate of discovery of new successful treatments than publicly-sponsored trials. However, time analysis indicated that the difference has disappeared over time, and that the success rates between industry sponsored and publicly sponsored trials have become comparable (Figure 11).

How can these results be explained? A fundamental aspect of any explanation must revolve around the issue of predictability of the results in advance and its implication for clinical trial system.

Our findings are consistent with several hypotheses, which are not necessary mutually exclusive:

1. the results represent ‘truth’. The observed higher success rates seen in industry-sponsored trials is rooted in the way commercial sponsors invest in drug development, so that only proposals with very promising data and high likelihood of success progress to RCT testing.[7] That is, the higher success rates seen in industry sponsored research is expected, and can be explained by extensive research and development efforts combined with multimillion dollars investment[23], intricate knowledge of the drugs, careful planning, and meticulous and professional execution, consistent with an associated objective of bringing a therapeutic agent to licensing approval and market.[7], [23], [24] Therefore, the results showing that the proportion of new treatments are superior to standard treatments is significantly higher in commercially sponsored trials is real and should not be taken as a surprise. [7], [24] The decrease in the differences in treatment success between publicly sponsored and industry sponsored over time may reflect increasing difficulties of developing ‘blockbuster’ drugs (such as onandenstron in palliative field, which accounted for a number of positive trials in this GSK cohort) which are typically associated with large effect sizes. As a result, the trials in later decades focused on detecting smaller treatment effects, which required larger sample sizes. Because it is more difficult to predict results in advance when effect sizes are smaller, the patterns of treatment success over time have been increasingly approaching HR or OR of no difference.
2. The results are artefact of flawed design or conduct of RCTs. The research over the last couple of decades have shown that the results of RCTs can frequently be explained by other factors such as differences in the data interpretation (‘spin’), the risk of bias, reporting bias, or choice of comparators. Although these factors may affect both industry sponsored and publicly sponsored trials[25], industry sponsored trials has been more often criticized for potentially biased research.[6], [26], [27] Research during the past decade has identified publication bias[28], [29], risk of bias[30], [31], the choice of control intervention[5], [32], or even interpretative ‘spin’[33] as key factors affecting trials' results. We believe that publication bias is unlikely as we had access to the summary reports about all trials (published and unpublished) conducted by GSK as well as CTG. Previous research has indicated that industry-sponsored trials are usually of better quality than publicly sponsored trials.[5], [6] In contrast with the previous research, we found that methodological quality of the CTG trials, with the exception of blinding, were better reported than in GSK trials. Specific information on blinding was more often described in GSK trials (82% compared to 35% of CTG trials). Details on allocation concealment were reported in all CTG trials, but only 14% of the GSK trials provided adequate description of allocation concealment. CTG trials more frequently reported all pre-specified outcomes (74%) compared to GSK trials (14%). However, the differences are likely a consequence of reporting and not actual conduct [34] as we could have obtained only summary instead of detailed research protocols in case of majority of GSK trials. The observed findings of our study were likely not affected by risk of bias. As shown in table 2, allocation concealment was inadequately reported in more RCT sponsored by GSK compared to CTG. However, the vast majority of RCTs conducted by GSK employed ‘blinding’, which typically demand and preserve adequate allocation concealment.[15] Most importantly, we detected no impact of risk of bias on the treatment effect. As shown in figures 6 and 7 there was no statistically significant test of interactions between the subgroups of trials with good compared to poor reporting. Therefore, the differences in the methodological quality is likely reflection of poor reporting than actual trial conduct.[34] The effect of choice of comparator deserves further discussion. As shown in Table 1, the GSK and CTG employed placebo/no therapy as a comparator in equal proportions. However, the success rates and the effect size in placebo-controlled trials were significantly higher in GSK than in CTG trials (Figures 8 and 9). The results favoured experimental treatments for all 3 metrics of treatment success (a proportion of statistically significant results favouring experimental treatment, according to the investigators' judgments and meta-analysis of data). This raises the question that the use of placebo did not reflect true uncertainty that RCTs are called to address.[17], [35] However, the results in the later placebo-controlled trials produced less significant effect indicating possible changes in the way placebo-controlled trials are designed. Whether this trend reflects changing philosophy toward the study design on the part of sponsor, regulators, or investigators, is not possible to discern from our data. The change in trend has coincided with the intensive scrutiny and criticism of industry sponsored trials with the call for reform.[27], [36] To the extent that our time analysis indicates the true trend, these changes in the clinical trial environment may be reflected in our observation of industry sponsored trials. ‘Spin’ was not likely explanation of our results as we followed a standardised methodology for data extraction and interpretation with high face and construct validity. As explained earlier, all data were independently extracted by two observers; in rare cases, where discrepancy occurred, the uniform consensus between the data extractors, and first two authors of the manuscript was easily achieved. Hence, it is unlikely that the ‘spin’ affected our data extraction and interpretation.
3. The results reflect differences in mix between the proportion of explanatory and pragmatic trials in industry compared to publicly sponsored trials.[37] Explanatory trials are focused on the proof of a concept, or mechanism, as for example, whether interventions works under ideal study circumstances (‘efficacy studies'). Pragmatic trials, on other hand, attempt to answer the question ‘which treatment (of already proven efficacy) is superior'; that is, which treatment will work better in a representative sample of patients to whom the study results will be extrapolated (‘effectiveness’ studies).[38] The effect size is expected to be much larger in explanatory than in pragmatic trials, which can explain the differences in the results between the GSK and CTG cohorts. In a retrospective study such as ours, it is impossible to clearly demarcate the pragmatic from explanatory trials. However, pragmatic trials typically do not use placebo[38]; pragmatic trials aim to compare the active therapy or even ‘best supportive care’ (no active therapy) in testing of one practical therapeutic strategy against the other. What one can deduce from the list is that the GSK rarely designed pragmatic trials, while this is rather common in the CTG cohort.
4. The results are coincidental. It is also possible that our observations are purely due to play of chance. There is no logical way refute this possibility outside of calling for future research to reproduce or disprove our findings.

Our data do not allow discerning between these hypotheses. In recent years, the existing system of clinical trials has become increasingly criticized for its inefficiencies with the calls for the substantial reform. The move for higher efficiency is largely based on demands to increase predictability of the results- also echoed in the proposals to replace traditional two-group parallel design with the ‘adaptive design’ to improve speed and the proportion of therapeutic discoveries.[39], [40]

The calls for increasing efficiency in clinical trial system should be contrasted with bioethical implications of the purported purpose of RCTs to address uncertainties about relative effects of competing interventions, which would require equipoise as a precondition for conduct of RCTs.[19], [41] We have previously postulated that the pattern of therapeutic discovery adheres to so called equipoise/uncertainty hypothesis.[3], [5], [11], [13], [17]–[19], [42] According to equipoise/uncertainty hypothesis, investigators cannot predict the effects of treatments in advance.[3], [17], [18], [43] Thus, sometimes new treatments will be superior, sometimes standard treatments will be more efficacious, and sometimes no differences will be detected. [3], [11], [17], [18], [43] Previous research analysing RCTs conducted between 1955–2006 by the US NCI-sponsored cooperative groups found that, depending on the metrics used, 25% to 50% of new cancer treatments are superior to standard treatments.[3] Very similar success rate was observed in the present CTG cohort and was also seen in the cohort of cancer trials conducted by the UK Medical Research Council[44], as well as in the cohort of non-cancer RCTs conducted by the UK government health technology assessment program[43] and the US National Institute of Neurological Disorders and Stroke.[45] Research synthesis of data from these four cohorts sponsored by the public funders indicates that conduct of publicly funded trials reflects the equipoise/uncertainty hypothesis.[13] In addition, remarkable reproducibility in the studies evaluating treatment success in publicly sponsored trials points out that once new treatments reach the stage of assessment in RCTs, the public can expect from its investment into clinical research a discovery rate of about 25% to 50%. According to the equipoise/uncertainty hypothesis [3], [5], [13], [17], [18], maintaining the unpredictability in results will help preserve the RCT system and if about half of new treatments are superior to standard treatments that should be considered a good ‘investment’.[3], [13], [17], [18] This also means that under this expectation of the probability of treatment success, most rational approach to testing of new treatment is to randomize patients between experimental vs. standard treatments.[17], [46] Indeed, when in equipoise, the majority of lay people[47] and members of Institutional Review Boards[48] accept randomization when there is an equal chance of allocation to successful treatment. In contrast, only 3% of people are willing to participate in RCTs when the probability of success of experimental treatment is 80∶20% or more.[47], [48] If there is higher likelihood that the conclusion of the study will be in favour of the sponsor's drugs, then conducting such a RCT would be ethically and scientifically questionable because, at least some people would be allocated to therapy that is believed to be inferior before the trial began and no valuable scientific information would emerge from such a study. Conceivably, this could also hinder participation of potential participants in RCTs, as most people would refuse randomization if chance of receiving better treatment is only 50% and the existing track record indicates that superior therapy could have been predicted in advance based solely on knowledge of sponsor.[17], [18]

An alternative view was expressed by Fries & Krishna.[7] Invoking the so called ‘design bias’ hypothesis, Fries & Krishnan argue that high predictability of the results in advance and violation of equipoise is essential to efficient medical progress, as it allows filtering of ineffective drugs early in the drug discovery process and identification of those drugs that are clinically useful and of positive societal value.[7] This view also appears to be supported by the recent writings of influential ethicists who have endorsed the normative view that equipoise is neither a necessary nor sufficient criterion for enrolment of patients into RCTs.[49] A wider societal debate is needed to resolve scientific, ethical and social benefits around the question of ‘optimal’ therapeutic success, which is direct function of predictability of results in advance.

A potential limitation of our analysis is that we have compared one industry sponsor with one academic clinical trials cooperative group. The design of our study has an important strength as it is the first one that relies on comparison of systematically constructed inception cohorts of trials from each sponsor. A limitation is whether the results are generalizable. While our data show that superiority of experimental treatments of CTG trials approximate that of other publicly sponsored trials[3], [43]–[45], we are not aware of published data that utilized inception cohort design from other industry sponsors. Nevertheless, our results are probably generalizable to other commercial sponsors, given that the GSK is the second largest pharmaceutical company in the world, and its approach to research is likely to be emulated by other smaller companies.

Regardless which type of research program- publicly sponsored compared to industry sponsored one favours, we believe that maintaining trust in RCTs at a societal level is greatly facilitated by providing potential clinical trial participants an accurate track record of treatment discoveries[50] _ENREF_40, which is one of the goals of this manuscript. While our results indicate high reproducibility of distribution of treatment success in publicly funded trials, future research is needed to address the hypotheses put forward here by assessing therapeutic outcomes in RCTs funded by other commercial sponsors utilizing the inception cohort design.

### Ethics

This study was approved by the University of South Florida Institutional Review Board (#100449).

## Supporting Information

Figure S1 **Method for Assessment of treatment success as per investigators judgments**
(TIF)

Figure S2 **Forest plot of sensitivity analysis for distribution of success rate for trials involving multiple comparisons (> 2 arms).** Trials in which more than one new treatment was compared to standard treatment by repeating the analysis using only one of the new intervention group favouring the experimental treatment (best case scenario), and use only the comparison favouring the standard arm (worst case scenario). The summary pooled estimate (odds/hazard ratio) is indicated by rectangles, with the lines representing 99% confidence intervals (CIs). * Represents a statistically significant test for interaction between subgroups. Although a formal test of interaction is statistically significant in the default analysis and best case scenario, the point estimates in best and worst case scenarios remains essentially the same as in the default analysis. The findings based on different assumptions of analysing multiple comparisons did not affect the results in any meaningful way.
(TIF)

Supplementary Material S1 **Additional explanations and results.**
(DOC)
