# Supplementary figures and images for: Treatment Success in Cancer: Industry Compared to Publicly Sponsored Randomized Controlled Trials
Source: PLoS One. 2013 Mar 21;8(3):e58711. doi: 10.1371/journal.pone.0058711 (PMC3605423; doi:10.1371/journal.pone.0058711)

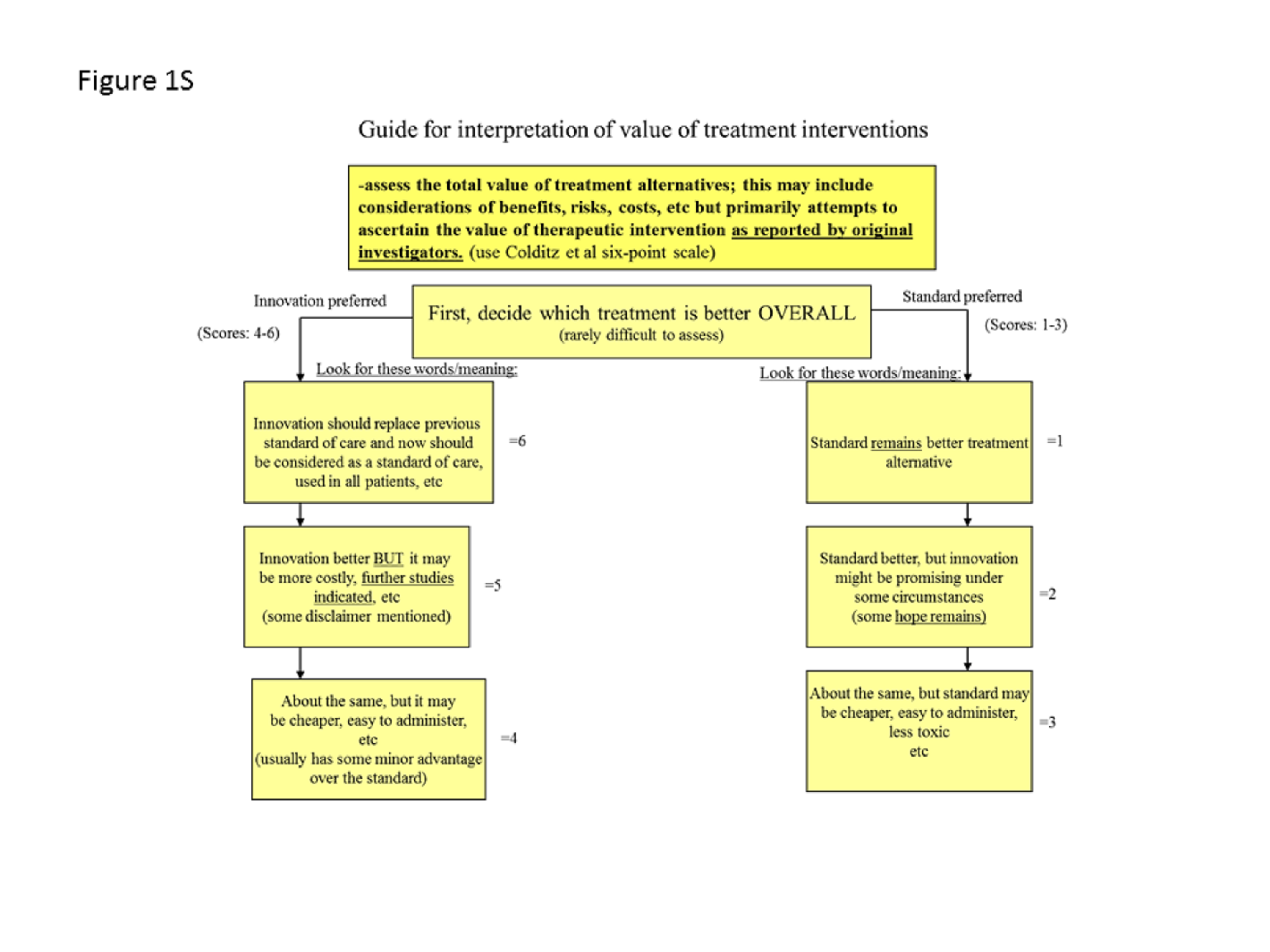

Supplement: Figure S1 — Method for Assessment of treatment success as per investigators judgments (TIF) [file pone.0058711.s001.tif]

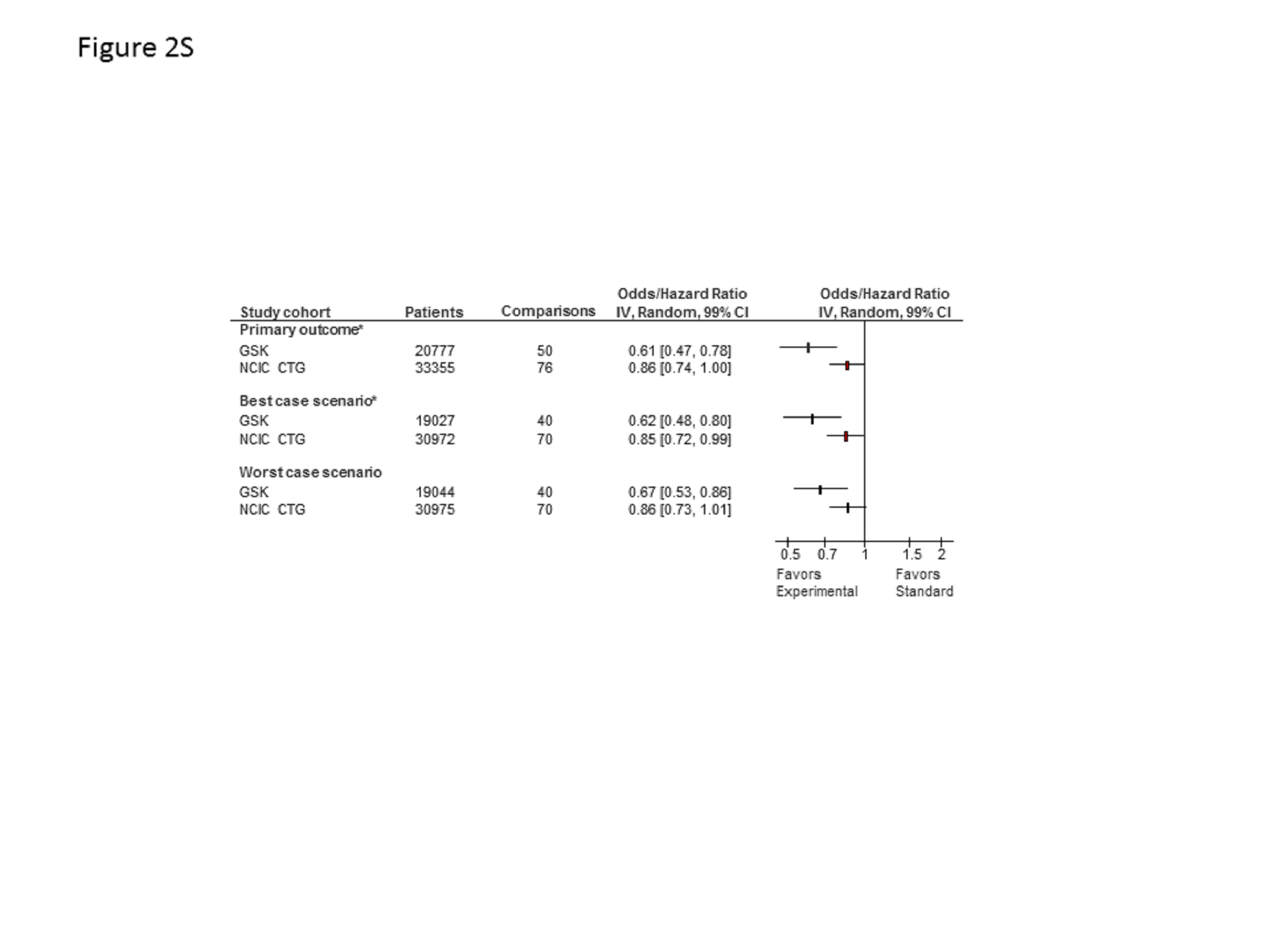

Supplement: Figure S2 — Forest plot of sensitivity analysis for distribution of success rate for trials involving multiple comparisons (> 2 arms). Trials in which more than one new treatment was compared to standard treatment by repeating the analysis using only one of the new intervention group favouring the experimental treatment (best case scenario), and use only the comparison favouring the standard arm (worst case scenario). The summary pooled estimate (odds/hazard ratio) is indicated by rectangles, with the lines representing 99% confidence intervals (CIs). * Represents a statistically significant test for interaction between subgroups. Although a formal test of interaction is statistically significant in the default analysis and best case scenario, the point estimates in best and worst case scenarios remains essentially the same as in the default analysis. The findings based on different assumptions of analysing multiple comparisons did not affect the results in any meaningful way. (TIF) [file pone.0058711.s002.tif]
