# Supplementary material for: Treatment Success in Cancer: Industry Compared to Publicly Sponsored Randomized Controlled Trials
Source: PLoS One. 2013 Mar 21;8(3):e58711. doi: 10.1371/journal.pone.0058711 (PMC3605423; doi:10.1371/journal.pone.0058711)
Supplement: Supplementary Material S1 — Additional explanations and results. (DOC) [file pone.0058711.s003.doc]

**Supplementary materials (S1):**

***Additional explanations and results***

*Categorization of NCIC CTG as publicly sponsored entity*

The NCIC Clinical Trials Group (Canada) is an academic cancer clinical trials cooperative group. The Group is funded through peer-review grants and contracts with industry. Its largest peer review grant is provided by the Canadian Cancer Society Research Institute. In its projects that involve collaborations with industry, including industry funding, the Group independently conducts its trials including oversight, management and analysis of trial data bases and publication of results. The NCIC CTG’s legal entity is Queen's University, Kingston, Ontario, Canada. The Group is not responsible to shareholders.

*Assessment of treatment success according to investigators judgment*

Assessment of treatment success as per investigators judgments was performed in two steps using the information provided in the discussion/conclusion section of the manuscript. In step one data abstractors used this information and first made a determination on whether experimental treatment was better than standard treatment or vice versa. That is, data abstractors assessed the total value of treatment alternatives which may include considerations of benefits, risks, costs, etc. but primarily attempts to ascertain the value of therapeutic intervention as reported by original investigators. In step 2 following the determination of superiority of experimental or standard treatment data abstractors searched for cues in the discussion/conclusion section to determine whether a score of 1 to 3 should be awarded if standard treatment was favoured or a score of 4-6 if experimental treatment was favoured. In case standard treatment was favoured a score of 1 was given if text such as “standard treatment remains better treatment alternative” was used. If investigators concluded that standard treatment is better but experimental treatment might have worked better in a sub-group of patients or under some circumstances a score of 2 was given. A score of 3 was given if authors concluded that there is no difference between standard and experimental treatment but the standard treatment was cheaper or easy to administer or less toxic. Using the same analogy a score of 4 was given if the experimental treatment had some minor advantage over the standard treatment. For example, if the experimental treatment was less toxic, cheaper or easy to administer a score of 4 was awarded. In cases where experimental treatment was considered better than standard but the results were not considered fully conclusive a score of 5 was given. For example, experimental treatment was better than standard but may be more expensive, or phrases such as “further studies needed” were used or experimental treatment was recommended with some disclaimers or caveats then a score of 5 was awarded. A score of 6 was given if authors clearly mentioned that new treatment should replace the standard of care. Figure 1S illustrates the method. This method has been used extensively both by us [3, 5, 11, 12, 13] and others [16] with high reproducibility. Our inter-rater agreement has consistently exceeded kappa of 0.85.
